# Strategies of maize silage supplementation of grazing dairy cows: Effects on milk production, pasture intake, grazing behaviour and methane emissions

**DOI:** 10.1016/j.dib.2024.110361

**Published:** 2024-03-22

**Authors:** Rémy Delagarde, Romain Guyard, Raphaël Boré, Benoît Rouillé

**Affiliations:** a PEGASE, INRAE, Institut Agro, 16 Le Clos, Saint-Gilles 35590, France; b IDELE, 42 rue Georges Morel, Beaucouzé 49071, France; c IDELE, Monvoisin, Le Rheu 35652, France

**Keywords:** Dairy cow, Precision livestock farming, Technology, Grazing

## Abstract

Supplementation strategy and grazing management can strongly influence dairy cow feeding behaviour, herbage intake, milk production and methane emissions. Two studies were conducted to investigate (1) the level of supplementation with partial mixed rations (PMR) and (2) the timing of maize silage feeding (morning vs. evening) for cows that have access to pasture either only during the day or day and night. A dataset was built that includes all individual cow measurements from both studies. It consists of 18 Microsoft® Excel files that correspond to several scales of information. The main file, “GrASTech_04_CowMeasurements”, contains individual weekly measurements of milk production and composition, body weight, supplement and herbage dry matter intake measured using the *n*-alkane method and grazing behaviour measured using Lifecorder Plus, for a total of 168 cow × week datapoints. Five Excel files provide supplementary information at larger scales: periods, experimental treatments, feeds offered and their chemical composition, pasture characteristics and grazing management, and cow characteristics. The remaining 12 Excel files provide information at the daily scale on weather (1 file), methane concentrations and emissions (1 file), the grazing schedule (1 file) and grazing behaviour (9 files). The files related to grazing behaviour include the daily pattern of grazing time every 2 min as determined by Lifecorder Plus, as well as the daily pattern of grazing time, rumination, overactivity, other activity, rest and standing every 5 min as determined by Feed'Live. This dataset can be used to better understand and investigate relations among and the influence of animal characteristics, grazing management, the supplementation strategy and weather conditions on daily herbage intake, grazing behaviour, milk production and methane emissions at a weekly scale. The detailed information on feeding and grazing behaviour can also be used to study between-cow and between-day variability in daily cow activities.

Specification Table

**Table:** 

Subject	Animal Science and Zoology
Specific subject area	Herbage intake, grazing behaviour and methane emissions of dairy cows as a function of the maize silage supplementation strategy
Type of data	Table
How data were acquired	Data were acquired at the INRAE experimental dairy farm of Méjusseaume (Le Rheu, Brittany, France, https://doi.org/10.15454/yk9q-pf68) from two experiments in spring 2021 and 2022. These experiments were part of the GrASTech project of the 2018 Joint Call of the Cofund ERA-Nets FACCE ERA-GAS (grant no. 696356), ICT-AGRI 2 (grant no. 618123) and SusAn (grant no. 696231). The first experiment investigated effects of the level of partial mixed ration (PMR) supplementation, for cows at pasture during the day and night. The second experiment investigated effects of the timing of maize silage supplementation when cows grazed either only during the day or during the day and night. Twenty-four lactating Holstein dairy cows were used in each experiment according to a 3 × 3 (Experiment 1) or 4 × 4 (Experiment 2) Latin square design. All measurements on cows were carried out individually at each period. Milk production was determined daily at each milking in a rotary milking system (DeLaval), milk composition by mid-infrared spectrophotometry, pasture intake by the *n*-alkane method using C_31_ and C_32_ as the internal and external markers, respectively, grazing behaviour thanks to two portable devices (Lifecorder Plus and Feed'Live), and methane emissions thanks to the Laser Methane Detector. At grazing, compressed sward height was measured with an electronic plate meter (Aurea Agrosciences), and herbage mass thanks to a motor scythe (Agria-Werke GmbH).
Data format	Microsoft® Excel files, with raw and analysed data
Parameters for data collection	The dataset contains all data measured during both experiments. Data on grazing characteristics and animal performance (i.e. intake and production) are provided at the weekly scale (mean values of the last week of each experimental period). Data on grazing behaviour and weather conditions are provided at the daily or minute scale to describe the daily pattern of cow activities and between-day relations between weather conditions and behaviour.
Description of data collection	The dataset includes raw and analysed data from two experiments conducted as part of the GrASTech project. It consists of 18 Microsoft® Excel files, each of which contains one kind of information: experimental periods, description of treatments, cow characteristics, cow measurements, herbage measurements, feed characteristics, weather, methane emissions, and the daily pattern of feeding and grazing activities.
Data source location	Experimental farm of PEGASE, INRAE, Institut Agro Méjusseaume, 35650 Le Rheu, Brittany, France
Data accessibility	Repository name: Recherche Data Gouv Data identification number: DOI Dataset GrASTech: 10.57745/GTTJAR Direct URL to data: https://doi.org/10.57745/GTTJAR

## 1 Value of the Data

• Published data on ruminants nutrition and behaviour at grazing are generally available only as adjusted means per treatment. This dataset combines *in vivo* cow measurements at the individual scale obtained under the same experimental conditions with the same measurement methods that require specific equipment and skills.
• The data may be useful for researchers and nutritionists who study dairy cow nutrition at grazing in order to simulate and/or predict intake regulation of individual cows. Few published studies have investigated different strategies of maize silage supplementation of grazing dairy cows that combine supplementation level and herbage allowance or combine the timing of supplement distribution and the daily access time to pasture [1,2,3].
• This study provides individual daily data on methane emissions estimated using the Laser Methane Detector [4,5]. These data can be used to study the variability and sources of variation in methane production, including characteristics of cows, feeds, herbage and pasture management.
• The dataset provides detailed information on cow behaviour at grazing, as cows were equipped simultaneously with two complementary devices (Lifecorder Plus and Feed'Live). It enables analysis of several types of behaviour at a precise scale (minute to hour), with a large dataset of 1245 and 2685 cow per day datapoints for Lifecorder Plus and Feed'Live, respectively. These analyses can be used to investigate multiple sources of variability in the feeding behaviour of grazing dairy cows.
• These *in vivo* experimental data can be used as an alternative or complementary approach to reduce future animal experimentation in the context of the ethical use of animals (the “Three Rs” principles).

## 2 Data Description

The GrASTech dataset consists of 18 Microsoft® Excel files: “GrASTech_01_Periods”, “GrASTech_02_Treatments”, “GrASTech_03_Cows”, “GrASTech_04_CowMeasurements”, “GrASTech_05_HerbageMeasurements”, “GrASTech_06_Feed”, “GrASTech_07_Weather”, “GrASTech_08_Methane”, “GrASTech_09_GrazingSchedule”, “GrASTech_10_LifecorderAccess”, “GrASTech_11_FeedLiveAccess”, “GrASTech_12_LifecorderGrazing”, “GrASTech_13_FeedLiveEating”, “GrASTech_14_”Ruminating, “GrASTech_15_Rest”, “GrASTech_16_Hyperactivity”, “GrASTech_17_OtherActivity” and “GrASTech_18_Standing”. Each file contains IDs that ensure interoperability of the tables. Each ID concatenates multiple codes in each file: the experiment (prefix “E”), the treatment within the experiment (prefix “T”), the cow within the experiment (prefix “C”) and the period within the experiment (prefix “P”) (Table 1).

**Table 1:** Descriptive statistics of the variables measured on cows in the “GrASTech_04_CowMeasurements” and in the “GrASTech_08_Methane” files of the dataset.

Variable	N	Mean	Standard deviation	Median	Minimum	Maximum
Body weight (kg)	168	606	45.6	604	508	716
Milk production (kg/day)	168	24.6	3.80	24.4	15.2	33.4
Milk fat concentration (g/kg)	168	37.2	3.74	37.5	27.7	46.3
Milk protein concentration (g/kg)	168	29.2	1.70	28.9	25.4	32.8
Milk fat production (g/day)	168	911	144.3	917	519	1286
Milk protein production (g/day)	168	717	104.2	703	456	945
Standardised milk production (kg/day)	168	23.3	3.40	23.4	14.0	32.1
Blood urea concentration (mg/L)	168	115	40.4	109	44	281
Milk urea concentration (mg/L)	164	108	44.0	108	13	264
Total intake (kg DM/day)	168	16.1	2.15	16.1	10.4	22.3
Herbage intake (kg DM/day)	168	11.8	2.58	11.8	5.0	18.7
Maize silage intake (kg DM/day)	168	4.0	1.97	4.7	0.0	7.0
Soya bean meal intake (kg DM/day)	168	0.2	0.36	0.0	0.0	1.2
Pasture access time (min/day)	168	956	341.5	1158	407	1217
Grazing time (min/day)^a^	168	423	62.9	427	271	540
Daily grazing time (min)^a^	168	285	47.7	279	196	407
Evening and night grazing time (min)^a^	168	138	93.4	178	0	279
First grazing bout duration (min)^a^	168	148	47.9	142	54	338
Number of grazing bouts (bouts/day)^a^	168	5.2	1.81	5.7	1.6	8.4
Herbage intake rate (g DM/min)	168	27.9	4.66	27.9	18.4	42.0
Mean peak methane conc. (ppm/m)^b^	668	108	45.0	100	29	306
Methane emission Chagunda (g/day)^c^	668	191	79.4	177	51	540
Methane emission Sorg (g/day)^d^	668	388	99.0	371	214	824

a From Lifecorder Plus.

b From Laser Methane Detector.

c From equation of [4].

d From equation of [5].

File 1, “GrASTech_01_Periods”, provides the following information, with one row per experiment per period (*N* = 7):

- Identification data (experiment, period)
- Start and end dates of the periods

File 2, “GrASTech_02_Treatments”, provides the following information, with one row per experiment per treatment (*N* = 7):

- Identification data (experiment, treatment)
- Characteristics of the treatments, which are defined by the type of supplement, the supplementation level, the access moment to the supplement, and the daily access time to pasture). In Experiment 1, three PMR (composed of 85 % maize silage and 15 % soyabean meal on a DM basis) supplementation levels were investigated. Cows received either 0, 4 or 8 kg DM/d of PMR, with access to this supplement 1 hour after evening milking and then 1 hour after morning milking, cows having access to pasture day and night (19 h/d). In Experiment 2, all cows received 5 kg DM/d of maize silage as supplement, and 4 feeding strategies were investigated. Two moments of access to this supplement were compared (after morning or after evening milking), in interaction with daily access time to pasture (only during the day, i.e. 7 h/d, or during day and night, i.e. 19 h/d).

File 3, “GrASTech_03_Cows”, provides the following information, with one row per experiment per cow (*N* = 48):

- Identification data (experiment, cow, group), with 2 experiments and 24 cows per experiment. Within an experiment, a group corresponds to a list of cows having followed exactly the same order of succession of treatments between periods. Group size was 4 cows in Experiment 1 and 6 cows in Experiment 2.
- Key dates (birth, calving) and lactation characteristics (lactation number, peak milk production) of each cow
- Cow characteristics during the 2-week reference period immediately before each experiment (body weight, body condition score, milk production, milk fat and protein concentrations, milk fat and protein production, standardised milk production)

File 4, “GrASTech_04_CowMeasurements”, provides the following information, with one row per cow per period and per experiment (*N* = 168):

- Identification data (experiment, group, cow, period, treatment, daily access time to pasture)
- Variables measured for cows during the last week of each experimental period (milk production, milk fat and protein concentrations, milk fat and protein production, standardised milk production, body weight, blood and milk urea, grazing behaviour (number of grazing bouts, duration of first grazing bout, grazing time), supplement and herbage intake, herbage intake rate)

File 5, “GrASTech_05_HerbageMeasurements”, provides the following information, with one row per experiment per period per treatment (*N* = 25):

- Identification data (experiment, period, treatment)
- Characteristics of pre-grazing herbage (sward bulk density, pre- and post-grazing sward height, herbage DM, ash, OM, N, CP, NDF and ADF concentrations, in vitro OM digestibility)
- Chemical composition of apparently selected herbage (DM, ash, OM, N, CP, NDF, ADF and ADL concentrations)

File 6, “GrASTech_06_Feed”, provides the following information, with one row per feed (*N* = 10):

- Feed identification data (experiment, period, feed type). Only supplement components are considered here, i.e. maize silage and soyabean meal, grazed herbage being described in File 5.
- Feed chemical composition (DM, ash, OM, N, CP, NDF, ADF and ADL concentrations)

File 7, “GrASTech_07_Weather”, provides the following information, with one row per day (*N* = 120):

- Day identification (experiment, date, day of the experiment, period)
- Weather data (rainfall; global radiation; mean daily minimum, maximum and mean temperatures; wind speed)

File 8, “GrASTech_08_Methane”, provides the following information, with one row per cow per day (*N* = 668):

- Identification (year, experiment, period, date, day of the experiment, cow, treatment)
- Mean peak methane concentration (Mean-peak-CH4, in ppm.m)
- Methane emissions (g/d) estimated using two published regressions, one from [4] (CH4-Chagunda), and the other from [5] (CH4-Sorg).

File 9, “GrASTech_09_GrazingSchedule”, provides the following information, with one row per day per treatment (*N* = 193):

- Day and treatment identification (period, day of the experiment, date, treatment)
- Time of day that cows entered and exited the paddocks
- Actual daily access time to pasture

Files 10 and 12, “GrASTech_10_LifecorderAccess” and “GrASTech_12_LifecorderGrazing”, respectively, provide the following information, with one row per day per cow (*N* = 1245):

- Identification (cow, day of the experiment, period, treatment)
- Time spent in the paddock (file 10) and grazing time (file 12) every 2 min. The time given corresponds to the end of the interval (e.g. 07:02:00 represents 07:00:00 to 07:02:00)

File 11, “GrASTech_11_FeedLiveAccess”, provides the following information, with one row per day per cow (*N* = 1273):

- Identification (cow, day of the experiment, period, treatment)
- Time spent in the paddock every 5 min. The time given corresponds to the end of the interval (e.g. 07:05:00 represents 07:00:00 to 07:05:00)

Files 13 to 18, “GrASTech_13_FeedLiveEating”, “GrASTech_14_Ruminating”, “GrASTech_15_Rest”, “GrASTech_16_Hyperactivity”, “GrASTech_17_OtherActivity”, “GrASTech_18_Standing”, respectively, provide the following information, with one row per day per cow (*N* = 2685):

- Identification (cow, day of the experiment, period, treatment)
- Time spent eating (file 13), in rumination (file 14), resting (file 15), in overactivity (file 16), in other activity (file 17) and standing (file 18) every 5 min. The time given corresponds to the end of the interval (e.g. 07:05:00 represents 07:00:00 to 07:05:00)

## 3 Experimental Design, Materials and Methods

Study 1 was conducted from 16 April to 18 June 2021 and study 2 from 22 April to 17 June 2022. In both experiments, cows were milked twice a day, at 7:00 and 16:00, and the grazing management was strip grazing, with access to a new strip of ungrazed pasture each morning.

### 3.1 Materials and methods for the experiments

#### 3.1.1 Cows, treatments, experimental design and conditions

##### Study 1

Three levels of supplementation (0, 4 or 8 kg DM/day of a partial mixed ration (PMR)) were compared according to a 3 × 3 Latin square design replicated eight times, balanced for potential residual effects of treatments, with 24 multiparous Holstein dairy cows in mid-lactation and three consecutive periods. The PMR was composed of maize silage and soya bean meal in an 85:15 ratio, on a DM basis. Each experimental period lasted 21 days: 2 days of feeding transition, followed by 10 days of adaptation and 9 days of measurements. Cows had access to pasture for 19 h/day (day and night grazing). Herbage allowance was 20, 17 or 14 kg DM/day at 4 cm above ground level for 0, 4 or 8 kg DM of supplement, respectively, to obtain a similar post-grazing sward height in all treatments.

##### Study 2

Four treatments were compared according to a 2 × 2 factorial design, with two feeding times of maize silage (morning or evening) and two daily access times to pasture (7 h/day, in the daytime between milkings, or 19 h/day, day and night grazing). Treatments were compared according to a 4 × 4 Latin square design replicated six times, balanced for potential residual effects of treatments, with 24 multiparous Holstein dairy cows in mid-lactation and four periods. Each experimental period lasted 14 days: 7 days of adaptation followed by 7 days of measurements. In all treatments, herbage allowance was 18 kg DM/day at 4 cm above ground level, and maize silage was fed individually at a rate of 5 kg DM/day. Contrary to study 1, there was no feeding transition between periods in study 2 because the diet composition was very similar between treatments. Periods were also shorter in study 2 than in study 1 for the same reason (14 vs 21 days).

#### 3.1.2 Measurements and analyses

##### Sward measurements

Pre- and post-grazing sward heights were measured daily using an electronic plate meter (30 × 30 cm, 4.5 kg/m², Aurea Agrosciences, Blanquefort, France), from ca. 50 readings per paddock following a W pattern on the strip to be grazed and just grazed, respectively. The pre-grazing pasture mass at 4 cm above ground level was estimated daily as the pre-grazing pasture height (minus 4 cm) measured previously times the sward bulk density. Sward bulk density was measured on d 5, 11, 13 and 19 in 2021 and d 5, 11 and 13 in 2022, by cutting 6 × 0.57 m strips (3 in 2021 and 2 in 2022) at 4 cm above ground level per treatment using an Agria 3600 motor scythe (Agria-Werke GmbH, Möckmühl, Germany). The pre- and post-cutting heights were measured in each strip using the same plate meter, with 12 measurements per strip. A 500-g subsample of fresh herbage per strip was oven-dried at 60 °C for 48 h to determine herbage DM concentration. The chemical composition of the offered herbage (> 4 cm) was determined from other 500-g subsamples taken on d 13 and 19 in study 1 and d 6 and 11 in study 2. The subsamples were washed with fresh water, oven-dried at 60 °C for 48 h, pooled per treatment and period, and then milled through a 0.8 mm screen for subsequent chemical analyses.

The botanical composition of the pastures was determined on d 13 of each period. Fifty handfuls (ca. 1 kg of fresh weight, in total) were collected following a W pattern on the total area of the paddock. The samples were manually sorted into four categories – grasses, clovers, dandelion and others – and each category was oven-dried at 60 °C for 48 h to determine its proportion on a DM basis. A 500-g sample of selected herbage was collected daily from each paddock by imitating the post-grazing sward height of the strip grazed the previous day, from d 14 to 21 in study 1 and d 8 to 14 in study 2. Samples were oven-dried at 60 °C for 48 h, then pooled by treatment and period, and milled through a 0.8 mm screen for chemical analyses.

##### Supplementation management and measurements

In study 1, PMR was distributed individually once a day, in the afternoon. Cows had individual access to PMR twice a day via individual troughs and electronic collars for one hour after the evening milking (from 16:30 to 17:30) and one hour after the morning milking (from 7:30 to 8:30). After this time, all PMR remaining in the trough was considered to be refusals. Refusals were removed and weighed for each cow once a day, in the morning. The DM concentration of the refusals was assumed to equal that of PMR distributed. In study 2, maize silage was fed either in the morning or in the evening depending on the treatment.

In both studies, a 500-g sample of maize silage was collected daily and oven-dried at 60 °C for 48 h to determine its DM concentration. Samples from d 17 to 20 in study 1 and d 10 to 13 in study 2 were pooled per period and milled through a 0.8 mm screen for chemical analyses. In study 1, soya bean meal was sampled once a week and oven dried at 60 °C for 48 h to determine its DM concentration. Samples from d 17 to 20 were pooled per period and milled through a 0.8 mm screen for subsequent chemical analyses.

##### Animal measurements

The body weight of cows was recorded automatically after each milking, thanks to a AWS100 DeLaval weight scale, with the Alpro software (DeLaval, Elancourt, France). Milk production was recorded per cow at each milking on a 28-place rotary milking system, with the Alpro software (DeLaval, Elancourt, France). Milk fat and protein concentrations were determined on d 18 to 21 in study 1 and d 11 to 14 in study 2, via mid-infrared spectrophotometry, using a Milkoscan instrument (Foss Electric, Hillerød, Denmark). Milk urea concentration was determined on d 19 and 21 in study 1 and d 13 and 14 in study 2, also via mid-infrared spectrophotometry.

Blood urea, non-esterified fatty acids and glucose concentrations were determined from blood samples collected in the morning of d 19 in study 1 and d 12 in study 2 via caudal venepuncture into vacutainers containing lithium heparin as an anticoagulant. After centrifugation (2 000 × *g* at 4 °C for 15 min), the plasma was stored at −20 °C prior to analyses.

Individual herbage intake was measured from d 17 to 21 in study 1 and d 10 to 14 in study 2 via the *n*-alkane method [6] using the ratio of herbage C_31_ (hentriacontane) to dosed C_32_ (dotriacontane). Cows were dosed after each milking with a cellulose stopper containing 340 mg of C_32_. Faecal grab samples were collected manually from the evening milking on d 17 to the morning milking on d 22 (d 10 to 15 in study 2). Faecal samples were stored at 4 °C before being pooled per period and per cow. Representative subsamples were oven-dried at 60 °C for 72 h and then milled through a 0.8 mm screen for subsequent chemical analyses to determine alkane concentrations. Alkane concentrations were also determined for samples of herbage apparently selected by the cows, maize silage and soya bean meal.

**Fig. 1:**
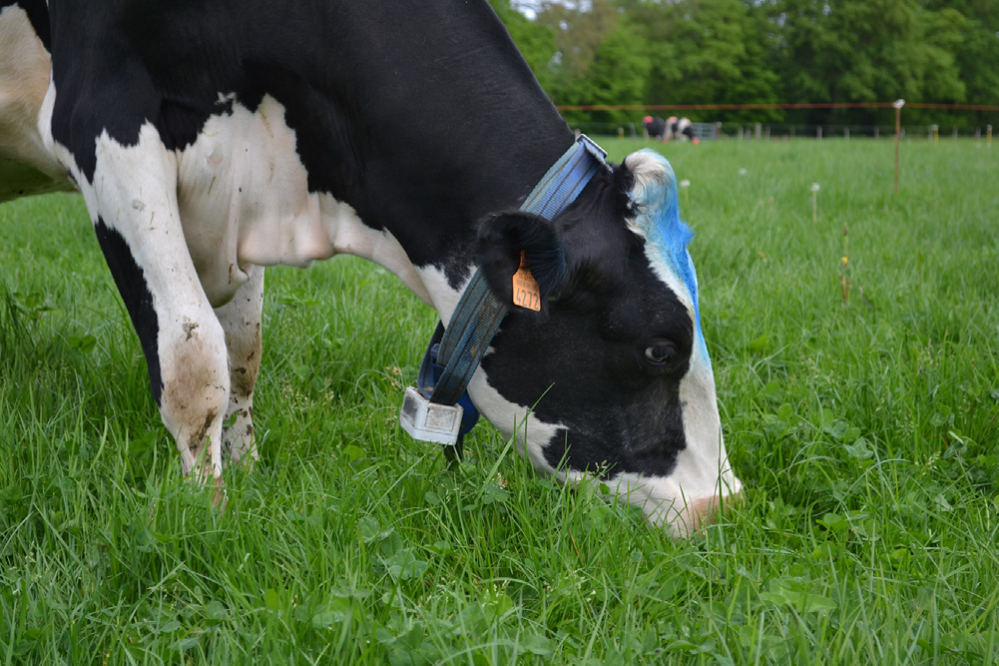
A cow equipped with the Lifecorder Plus device.

Grazing behaviour was recorded individually using the Lifecorder Plus (Kenz, Suzuken Co. Ltd., Nagoya, Japan). This device is small, weighs 50 g, with dimensions of 70 × 40 × 25 mm. It contains a uniaxial accelerometer that records, for 2-min periods, the average activity level detected at 4-s intervals according to 11 scaled magnitudes: 0 (none), 0.5 (subtle) and 1 to 9 (light to vigorous, respectively) [7,8]. The activity level of a cow engaged in a non-grazing activity, such as rumination, is close to 0 [7]. Its accuracy is very high, with a mean prediction error at the cow per day scale of only 5 %, with no mean bias or line bias, and with no confusion with other activities such as rumination or resting [7] Therefore, the Lifecorder Plus is suitable for recording only grazing activity, not indoor eating of supplements, rumination or other activity. Each Lifecorder Plus was placed in a waterproof box (90 × 60 × 55 mm, Fig. 1), which was attached to a collar placed around the cow's neck. Cows were fitted from d 13 to 21 in study 1 and d 7 to 14 in study 2. Halfway through each measurement period (d 18 in study 1 and d 11 in study 2), the Lifecorder Plus devices were switched between cows to ensure that all cows had at least several full-day records in case a device encountered a problem. On average, there were 7.4 ± 1.00 valid full-day recordings per cow and period, with a minimum of 3, and a maximum of 8. Data were downloaded at the end of each period, and the extracted files contained the average activity level (range: 0 to 9) recorded every 2 min. A grazing sequence (i.e. meal) was defined as a period of at least 6 min during which the level of activity exceeded 0.5, this value of threshold minimising the mean prediction error when compared to visual observations [7,9]. Short intra-meal periods of 2 or 4 min (e.g. periods of no activity during a meal) were considered to be grazing [10], but short activity periods of 2 or 4 min were not [3].

Each cow was permanently equipped with a Feed'Live (Medria, Saint-Lô, France) in both studies, including during days of transition and adaptation, except during the 3 first days of study 2. This device, which contains a triaxial accelerometer, is an activity logger attached to a collar placed around the cow's neck. It records standing or lying activity and six different behaviours: indoor eating, grazing, rumination, resting, overactivity and unidentified activities (hereafter, “other activity”) [11]. Activity is recorded as the cow's dominant activity during 5-min periods [11]. Data were automatically transferred from a transmitter in the collars to an antenna in the barn. Data were then processed by New Medria (Châteaubourg, France), and processed files were extracted from New Medria's website. Activity classified as indoor eating when cows were at pasture was considered to be grazing.

The methane concentration of the air inhaled and exhaled by each cow was measured using the LaserMethane mini-g (LMD, Tokyo Gas Engineering Solutions, Japan) [4]. Measurements were taken from d 11 to 14 of each period. Each cow was measured twice a day, once in the morning and once in the afternoon, in a pre-defined random order, by the same trained operator each year. During each measurement, the operator stood 2 m away from one side of the cow and pointed the device's laser beam perpendicularly at one of its nostrils. The operator then had to maintain the laser beam's distance from and angle to the nostril while following the cow for 4 min to validate the measurement. The distance between the LMD and the cow was controlled thanks to a laser meter attached to the LMD. The device measures methane concentration every 0.5 s. The mean of peaks methane concentration per cow (Mean-peak-CH4, in ppm.m) was calculated for each 4-min measurement period, and then per cow and per day, as by [4]. Methane emissions per cow (CH4-Chagunda or CH4-Sorg, in g/d) were then calculated in two ways using the following equations Eq. 01 from [4] and Eq. (2) from [5], respectively.

(1) CH4−Chagunda=Mean−peak−CH4×TV×p(CH4)×D

with TV the tidal volume (3100 mL/s), p(CH4) the density of methane (0.000657 mg/L) and D the duration of a day, in seconds (86,400 s).

(2) CH4−Sorg=Mean−peak−CH4×2.2(±0.07)+150(±22.1)

##### Chemical analyses

Ash was determined via calcination in a muffle furnace at 550 °C for 8 h [12]. Nitrogen concentration was measured via the Dumas method [12] using a Leco instrument (Leco Corp., St. Joseph, MI, USA). Pepsin-cellulase digestibility of herbage was determined according to [13]. The concentrations of NDF, ADF and ADL were measured according to [14] using a Fibersac extraction unit (Ankon Technology Corp., Fairport, NY, USA). The *n*-alkanes concentrations were determined according to [6] following direct saponification [15]. Blood urea, NEFA and glucose concentrations were measured using a multi-parameter analyser (KONE Instruments 200 Corporation, Espoo, Finland).

### 3.2 Materials and methods for building the dataset

#### 3.2.1 Data recovery

The dataset was built using all available data, whose scale depended on the type of measurement. Data related to pasture management and measurements, milk production, individual herbage and supplement intake, blood metabolites and feeding behaviour were acquired, processed (except for Feed'Live raw data processed by New Medria), calculated and provided by the co-authors from INRAE (e.g. R. D. and R. G.). Data related to methane emissions were acquired, processed, calculated and provided by the co-authors from IDELE (R. B. and B. R.). Data related to weather conditions (i.e. rainfall, air temperature, wind speed) were measured hourly at an INRAE weather station 500 m from the experimental paddocks. Weather data were monitored from the INRAE CLIMATIK platform (https://intranet.inrae.fr/climatik/, in French), managed by the AgroClim laboratory in Avignon, France. Weather data were downloaded as mean values per day.

#### 3.2.1 Data calculation

Standardised milk production (SMP, kg/day), which equals milk production (M, kg/day) corrected to 40 and 31 g/kg for milk fat (F) and true protein (P) concentrations, respectively, was calculated according to Eq. (3) [16]:

(3) SMP=[MP×(0.42+[0.0053×(F−40)]+[0.0032×(P−31)])]/0.42

Grazing time during the day was calculated as the sum of grazing activities between morning and evening milkings. Grazing time during the evening and night was calculated as daily grazing time minus grazing time during the day. Herbage intake rate (in g DM/min) was calculated for each cow × period as herbage intake (g DM/day) divided by the mean grazing time (min/day) provided by the Lifecorder Plus.

## Ethics Statement

The studies were conducted following French and European Union legislation on animal experimentation and animal welfare. All procedures related to the care and management of animals were approved by an ethics committee of the French Ministry of Agriculture (CREEA, Comité Rennais d'Ethique en matière d'Expérimentation Animale, No. 07), in agreement with French regulations (decree 2001-464, 29 May 2001) (approval number: APAFIS #24101-2020021220597961 v2).

## Funding

The authors acknowledge financial support from the partners of the 10.13039/1000124272018 Joint Call of the Cofund ERA-Nets FACCE ERA-GAS (grant no. 696356), 10.13039/501100003621ICT-AGRI 2 (grant no. 618123) and 10.13039/100009634SusAn (grant no. 696231).

## Data Availability

Maize silage supplementation in grazing dairy cows: milk production, pasture intake, behaviour and methane emissions (Original data) (Dataverse).

## References

[1] Civiero M., Delagarde R., Berndt A., Rosseto J., de Souza M.M., Schaitz L.H., Ribeiro Filho H.M.N. (2021). Progressive inclusion of pearl millet herbage as a supplement for dairy cows fed mixed rations: effects on methane emissions, dry matter intake, and milk production. J. Dairy Sci..

[2] Ribeiro Filho H.M.N., Dall-Orsoletta A.C., Mendes D., Delagarde R. (2021). Dry matter intake and milk production of grazing dairy cows supplemented with corn silage or a total mixed ration offered ad libitum in a subtropical area. Anim. Sci. J..

[3] Miguel M.F., Ribeiro-Filho H.M.N., Delagarde R. (2023). Effects of corn silage supplementation strategy and grazing intensity on herbage intake, milk production, and behavior of dairy cows. J. Dairy Sci..

[4] Chagunda M.G.G., Ross D., Roberts D.J. (2009). On the use of a laser methane detector in dairy cows. Comput. Electron. Agric..

[5] Sorg D., Difford G.F., Mühlbach S., Kuhla B., Swalve H.H., Lassen J., Strabel T., Pszczola M. (2018). Comparison of a laser methane detector with the GreenFeed and two breath analysers for on-farm measurements of methane emissions from dairy cows. Comput. Electron. Agric..

[6] Mayes R.W., Lamb C.S., Colgrove P.M. (1986). The use of dosed and herbage n-alkanes as markers for determination of herbage intake. J. Agric. Sci..

[7] Delagarde R., Lamberton P. (2015). Daily grazing time of dairy cows is recorded accurately using the Lifecorder Plus device. Appl. Anim. Behav. Sci..

[8] Ueda Y., Akiyama F., Asakuma S., Watanabe N. (2011). The use of a physical activity monitor to estimate the eating time of cows in pasture. J. Dairy Sci..

[9] Lemoine M., Piriou M., Charpentier A., Delagarde R. (2021). Validation of the Lifecorder Plus device for accurate recording of the grazing time of dairy goats. Small Rumin. Res..

[10] Gibb M.J., Keane M.G, O'Riordan E.G. (1998). Pasture Ecology and Animal Intake.

[11] Fischer A., Delagarde R., Faverdin P. (2018). Identification of biological traits associated with differences in residual energy intake among lactating Holstein cows. J. Dairy Sci..

[12] Association of Official Analytical Chemists (AOAC) (2019).

[13] Aufrère J., Michalet-Doreau B. (1988). Comparison of methods for predicting digestibility of feeds. Anim. Feed Sci. Technol..

[14] Van Soest P.J., Robertson J.B., Lewis B.A. (1991). Methods for dietary fibre, neutral detergent fibre, and nonstarch polysaccharides in relation to animal nutrition. J. Dairy Sci..

[15] Vulich S.A., O'Riordan E.G., Hanrahan J.P. (1991). Effect of litter size on herbage intake at pasture by ewes and their progeny. Anim. Prod..

[16] INRA (French National Institute for Agricultural Research) (2018).

